# Supplementary material for: Splice-Junction-Based Mapping of Alternative Isoforms in the Human Proteome
Source: Cell Rep. Author manuscript; Available in PMC 2020 Jan 15. (PMC6961840; doi:10.1016/j.celrep.2019.11.026)
Supplement: 2 [file NIHMS1546469-supplement-2.zip › DF1/PXD009737/TestisGluC/TestisGluC_25_MAP4_AGQNDPLKDSYVPLE.pdf]

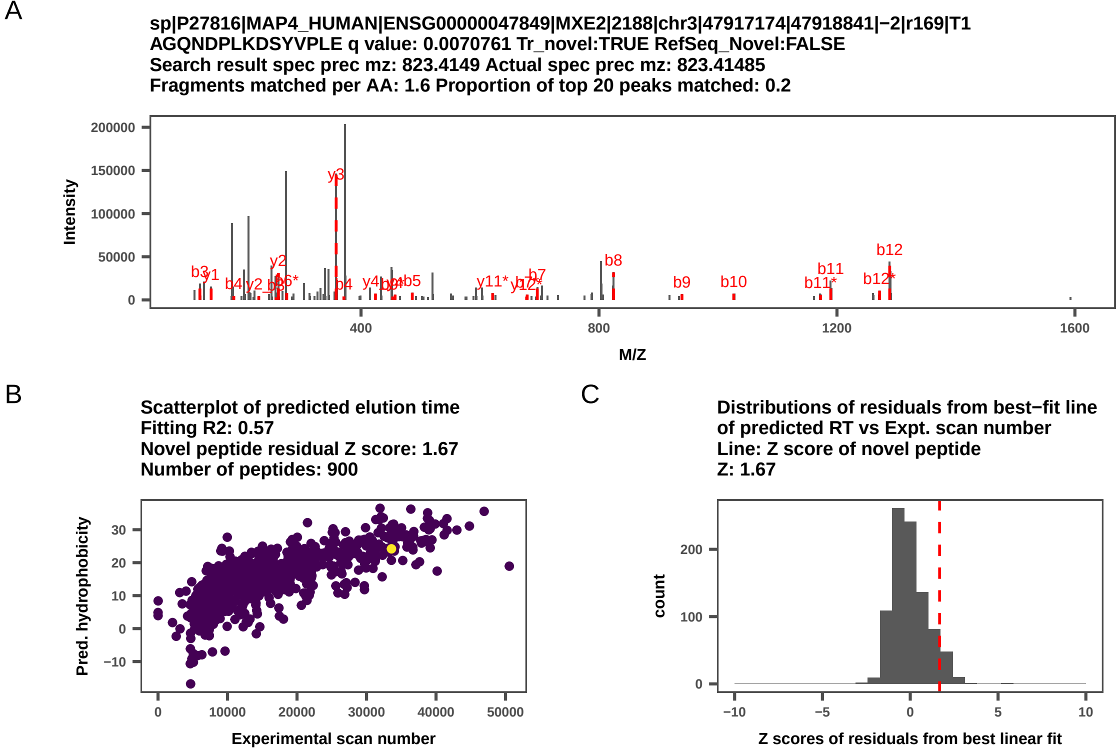
A
sp|P27816|MAP4_HUMAN|ENSG00000047849|MXE2|2188|chr3|47917174|47918841|−2|r169|T1
AGQNDPLKDSYVPLE q value: 0.0070761 Tr_novel:TRUE RefSeq_Novel:FALSE
Search result spec prec mz: 823.4149 Actual spec prec mz: 823.41485
Fragments matched per AA: 1.6 Proportion of top 20 peaks matched: 0.2
Intensity
200000
150000
100000
50000
0
y3
y2
b8
b9
b10
b11
b11*
b12
b12*
400
800
1200
1600
M/Z
B
Scatterplot of predicted elution time
Fitting R2: 0.57
Novel peptide residual Z score: 1.67
Number of peptides: 900
Pred. hydrophobicity
30
20
10
0
−10
0
10000
20000
30000
40000
50000
Experimental scan number
C
Distributions of residuals from best−fit line
of predicted RT vs Expt. scan number
Line: Z score of novel peptide
Z: 1.67
count
200
100
0
−10
−5
0
5
10
Z scores of residuals from best linear fit
